# Metagenomic analysis and metabolite profiling of deep–sea sediments from the Gulf of Mexico following the Deepwater Horizon oil spill

**DOI:** 10.3389/fmicb.2013.00050

**Published:** 2013-03-15

**Authors:** Nikole E. Kimes, Amy V. Callaghan, Deniz F. Aktas, Whitney L. Smith, Jan Sunner, BernardT. Golding, Marta Drozdowska, Terry C. Hazen, Joseph M. Suflita, Pamela J. Morris

**Affiliations:** ^1^Baruch Marine Field Laboratory, Belle W. Baruch Institute for Marine and Coastal Sciences, University of South CarolinaGeorgetown, SC, USA; ^2^Department of Microbiology and Plant Biology, University of OklahomaNorman, OK, USA; ^3^Institute for Energy and the Environment, University of OklahomaNorman, OK, USA; ^4^School of Chemistry, Newcastle UniversityNewcastle upon Tyne, UK; ^5^Department of Civil and Environmental Engineering, University of TennesseeKnoxville, TN, USA; ^6^Department of Microbiology, University of TennesseeKnoxville, TN, USA; ^7^ Department of Earth and Planetary Sciences, University of TennesseeKnoxville, TN, USA; ^8^Ecology Department, Lawrence Berkeley National LaboratoryBerkeley, CA, USA

**Keywords:** Deepwater Horizon, metagenomics, metabolomics, oil-degradation

## Abstract

Marine subsurface environments such as deep-sea sediments, house abundant and diverse microbial communities that are believed to influence large-scale geochemical processes. These processes include the biotransformation and mineralization of numerous petroleum constituents. Thus, microbial communities in the Gulf of Mexico are thought to be responsible for the intrinsic bioremediation of crude oil released by the Deepwater Horizon (DWH) oil spill. While hydrocarbon contamination is known to enrich for aerobic, oil-degrading bacteria in deep-seawater habitats, relatively little is known about the response of communities in deep-sea sediments, where low oxygen levels may hinder such a response. Here, we examined the hypothesis that increased hydrocarbon exposure results in an altered sediment microbial community structure that reflects the prospects for oil biodegradation under the prevailing conditions. We explore this hypothesis using metagenomic analysis and metabolite profiling of deep-sea sediment samples following the DWH oil spill. The presence of aerobic microbial communities and associated functional genes was consistent among all samples, whereas, a greater number of Deltaproteobacteria and anaerobic functional genes were found in sediments closest to the DWH blowout site. Metabolite profiling also revealed a greater number of putative metabolites in sediments surrounding the blowout zone relative to a background site located 127 km away. The mass spectral analysis of the putative metabolites revealed that alkylsuccinates remained below detection levels, but a homologous series of benzylsuccinates (with carbon chain lengths from 5 to 10) could be detected. Our findings suggest that increased exposure to hydrocarbons enriches for Deltaproteobacteria, which are known to be capable of anaerobic hydrocarbon metabolism. We also provide evidence for an active microbial community metabolizing aromatic hydrocarbons in deep-sea sediments of the Gulf of Mexico.

## INTRODUCTION

The Deepwater Horizon (DWH) blowout resulted in the largest marine US oil spill to date, in which 4.1 million barrels of crude oil flowed into the depths (~1500 m) of the Gulf of Mexico (Operational Science Advisory Team, 2010). Although an estimated 78% of the oil was depleted through either human intervention or natural means by August 2010 (Ramseur, 2010), the fate of the remaining 22% was uncertain. Evidence subsequently showed that both oil (Hazen et al., 2010; Mason et al., 2012) and gas (Kessler et al., 2011) persisted in the Gulf of Mexico water column, affecting deep-sea (>1000 m) microbial communities that potentially facilitate the biodegradation of residual hydrocarbons. Much less is known about the impact of anthropogenic hydrocarbons on the microbial communities of deep-sea sediments. Although much of the hydrocarbons from sub-sea oil spills and natural seeps may rise to the surface, there are water-soluble components in oil as well as hydrocarbons adhering to solid particulates that can settle in deep-sea sediments (Ramseur, 2010). After the 1979 Ixtoc I oil spill, for example, in which over three million barrels of oil flowed into the Gulf of Mexico, it is estimated that 25% of the oil was transported to the sea floor (Jernelov and Linden, 1981).

The deep-sea biosphere, including deep-sea sediments, is both one of the largest and one of the most understudied ecosystems on earth (Jøgensen, 2011). Although the global estimates of prokaryotic biomass supported by deep-subsurface sediments are lower than originally thought, regional variation supports the presence of abundant and diverse sub-seafloor microbial communities in continental shelf areas, such as the Gulf of Mexico (Kallmeyer et al., 2012). This is especially true for the more surficial sediment communities, such as those utilized in this study. Evidence suggests that these deep-sea sediment communities support diverse metabolic activities (D’Hondt et al., 2004, 2009), including evidence of hydrocarbon degradation in microbial communities associated with cold water hydrocarbon seeps located in the Gulf of Mexico (Joye et al., 2004; Lloyd et al., 2006, 2010; Orcutt et al., 2010). As a result, it has been suggested that the microbial communities in the Gulf of Mexico deep-sea sediment would play a role in the biodegradation of persistent oil components following the DWH blowout. Despite numerous advances pertaining to individual microorganisms capable of metabolizing hydrocarbon compounds (Seth-Smith, 2010) and community responses to natural hydrocarbon seeps (Lloyd et al., 2010; Orcutt et al., 2010), little is known about the microbial capacity for oil-degradation within deep-sea sediment communities under the circumstances presented by the DWH spill, including the extreme depth (~1500 m) and the sudden hydrocarbon exposure.

To gain a better understanding of the sediment-associated microbial response to the DWH oil spill, deep-sea sediment cores were collected by a Lawrence Berkeley National Laboratory (LBNL) team aboard the R/V Gyre in the area surrounding the DWH oil spill between September 19 and October 10, 2010. Preliminary chemical analysis revealed that the cores closest to the DWH spill contained high levels of polycyclic aromatic hydrocarbons (PAHs; >24,000 μg/kg) compared to distant cores (~50 μg/kg), confirming a greater exposure of the resident microflora to aromatic hydrocarbons near the DWH well (Operational Science Advisory Team, 2010). Although it is likely that the DWH oil spill contributed to the higher PAH levels observed, other sources that could have influenced these levels include natural seeps located near the DWH site and drilling fluids.

In this study, we hypothesized that increased hydrocarbon exposure results in the alteration of microbial community structure, such that it reflects the selection for organisms capable of the anaerobic metabolism of petroleum constituents. We performed metagenomic sequencing on three of the deep-sea sediment samples collected by LBNL (described above) and compared our results to a Gulf of Mexico deep-subsurface sediment metagenomic library sequenced prior to the DWH oil spill (Biddle et al., 2011). To complement the metagenomic analysis, metabolic profiling was used to detect homologous series of putative signature metabolites associated with anaerobic hydrocarbon biodegradation. Our data indicated significant differences among the microbial communities examined in this study compared to those detected prior to the DWH oil spill. Moreover, the metabolite profiling revealed significantly more putative metabolites in the two samples closest to the DWH site relative to the more distant background site. These findings were consistent with the metagenomic data showing an increase in the number of functional genes associated with anaerobic hydrocarbon degradation in samples closest to the DWH.

## MATERIALS AND METHODS

### SAMPLE COLLECTION

**FIGURE 1:**
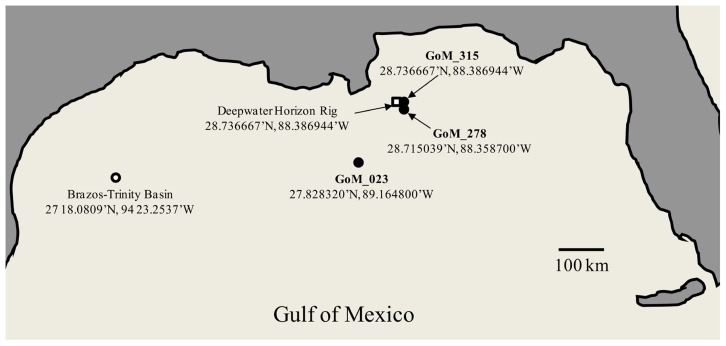
**Map of the Gulf of Mexico sampling sites.** Open square – DWH rig; filled circle – sampling sites from the current study; open circle – BT Basin sampling site from Biddle et al. (2011). The Peru Margin (PM) sampling sites used for comparison are described in Biddle et al. (2008).

Deep-sea sediment cores were collected by LBNL from the area surrounding the DWH oil spill in the Gulf of Mexico during six cruises by the R/V Gyre from September 16 to October 20, 2010 (Operational Science Advisory Team, 2010). An OSIL Mega corer (Bowers and Connelly) was used to collect deep-sea sediment cores, and overlying water was siphoned off using a portable peristaltic pump. The capped sediment cores were frozen at -80°C and shipped on dry ice to the LBNL where the cores were sectioned while frozen. The three cores utilized in this study were designated SE-20101017-GY-D040S-BC-315 (GoM315); SE-20101017-GY-D031S-BC-278 (GoM278); and SE-20100921-GY-FFMT4-BC-023 (GoM023). GoM315 and GoM278 were located near the DWH well (0.5 and 2.7 km, respectively), while GoM023 was located at a distance of 127 km from the DWH well (**Figure 1**). One-half of each core (GoM315, GoM278, and GoM023), approximately 5^′^^′^ diameter and 1^′^^′^ thick, was sent on dry ice to the University of South Carolina Baruch Marine Field Laboratory in Georgetown, SC, USA. Upon arrival they were further subsectioned in half using sterile razorblades in a biosafety hood. One half was used for DNA extraction and metagenomic analysis, while the other half was sent on dry ice to the University of Oklahoma (Norman, OK, USA) for metabolomic analysis.

### DNA EXTRACTION

Inside a biosafety hood, a sterile razor blade was used to cut a 3–4 g wedge from each of the three frozen cores (GoM315, GoM278, and GoM023). Community DNA was extracted from each core using a PowerMax^®^ Soil DNA Isolation kit (Mo Bio Laboratories, Inc., Carlsbad, CA, USA) according to the manufacturer’s instructions. The resulting DNA (~ 2 μg) from each sample was purified and concentrated via ethanol precipitation. The quality and quantity of the DNA were assessed via gel electrophoresis on a 2% agar gel with a 1 kb ladder and spectrophotometer analysis.

### METAGENOMIC SEQUENCING AND ANALYSIS

Approximately 1 μg DNA (per core sample) was sent to Engencore (University of South Carolina, Columbia, SC, USA), where high-throughput sequencing was performed using the Roche 454 FLX pyrosequencing platform. The sequencing results were recorded as SFF files and uploaded to the MetaGenome Rapid Annotation Subsystems Technology (MG-RAST) server for analysis (Meyer et al., 2008). Each file underwent quality control (QC), which included quality filtering (removing sequences with ≥5 ambiguous base pairs), length filtering (removing sequences with a length ≥2 standard deviations from the mean), and dereplication (removing similar sequences that are artifacts of shotgun sequencing). Organism and functional identifications were made using a BLAT [Basic Local Alignment Search Tool (BLAST)-like alignment tool] search of the integrative MG-RAST M5NR database, which is a non-redundant protein database that combines sequences from multiple common sources. All identifications were made using a maximum e-value of 1e-5, a minimum identity cutoff of 50%, and a minimum alignment length of 50 bp. The hierarchical clustering/heat map comparisons were constructed in MG-RAST using dendrograms based on abundance counts for each category examined. Similarity/dissimilarity was determined using a Euclidean distance metric, and the resulting distance matrix was combined with ward-based clustering to produce dendrograms. Diversity indices for species richness and diversity estimates were calculated using EstimateS software (Colwell, 2006). Circular recruitment plots were created through the comparison of each metagenomic library to the whole genomes of reference organisms (Refseq genomes only) using a maximum e-value of 1e-5 and a log10 abundance scale. Three organisms of interest were investigated: *Alcanivorax borkumensis* SK2 (Yakimov et al., 1998; Schneiker et al., 2006; dos Santos et al., 2010), an aerobic gammaproteobacterium that utilizes oil hydrocarbons as its exclusive source of carbon and energy and is often the most dominant bacterium in oil-polluted marine systems (Harayama et al., 1999; Kasai et al., 2001; Hara et al., 2003; Yakimov et al., 2005), *Desulfatibacillum alkenivorans *AK-01, a sulfate-reducing, *n*-alkane and *n*-alkene utilizing Deltaproteobacterium (So and Young, 1999; Callaghan et al., 2012), and *Geobacter metallireducens *GS-15, a metal-reducing, aromatic hydrocarbon utilizer within the *Deltaproteobacteria *(Lovley et al., 1993).

### PCR AMPLIFICATION OF FUNCTIONAL GENES

Sediment DNA from GoM315, GoM278, GoM023 was also interrogated with nine primer set combinations specific to *assA* and/or *bssA* (Callaghan et al., 2010). The *assA* and *bssA* genes encode the catalytic subunits of the anaerobic glycyl radical enzymes, alkylsuccinate synthase (ASS; also known as methylalkylsuccinate synthase, MAS; Callaghan et al., 2008; Grundmann et al., 2008) and benzylsuccinate synthase (BSS; Leuthner et al., 1998), respectively. Polymerase chain reaction (PCR) SuperMix (2X Dreamtaq, Fermentas) was used to set up 50-μL reactions containing 25 μL of 2X Dreamtaq mastermix, 0.4 μM of each primer, 5 μL of betaine (5 M stock), and 10 ng of DNA template. A modified touchdown PCR method (Muyzer et al., 1993) was used to minimize unspecific amplification. The cycling program was as follows: 95°C for 4 min followed by 2 cycles at each annealing temperature (i.e., 95°C for 1 min, 63–52°C for 1 min, 72°C for 2 min), 19 cycles at the plateau annealing temperature (53°C), and a final extension step at 72°C for 10 min.

### CONSTRUCTION AND PHYLOGENETIC ANALYSIS OF *assA* AND *bssA* CLONE LIBRARIES

Polymerase chain reaction products were purified using the Qiaquick purification kit (Qiagen) and cloned into either pCRII or pCRII-TOPO vector (Invitrogen, Carlsbad, CA, USA) following the manufacturer’s instructions. For each PCR product, colonies were picked into individual wells of two 96-well microtiter plates and grown overnight. Inserts of the correct size were sequenced using the M13R priming site. After sequencing, reads were trimmed to remove vector and primer sequences before further analysis. Sequences from each respective library were assembled into operational taxonomic units (OTUs) of ≥97% sequence identity using Lasergene 7.2 (DNASTAR Inc., Madison, WI, USA). The *assA*/*bssA* OTUs were aligned with *assA* and *bssA* genes from described strains for which complete sequences were available and the best BLAST matches National Center for Biotechnology Information (NCBI). Neighbor-joining trees were constructed in MEGA4 (Kumar et al., 2008) using the Tajima–Nei distance method, with pairwise deletion and performing 10,000 bootstrap replicates. The glycyl radical enzyme, pyruvate formate lyase (PFL), served as the outgroup. The DNA sequences of GoM *assA* and *bssA* OTUs were deposited in GenBank under the accession numbers JX135105 through JX135128.

### METABOLOMIC EXTRACTIONS AND ANALYSIS

Approximately 25 g of each core sample was thawed in 20 mL of double-distilled sterile water and then acidified with 10 N HCl until the pH was ≤2. Each sample was mixed with 100 mL of ethyl acetate and stirred overnight. The water phase was removed and the ethyl acetate solution was dried over anhydrous Na_2_SO_4_, concentrated by rotary evaporation to approximately 2 mL and reduced further under a stream of N_2_ to a volume of 100 μL. Half of the extract was derivatized and analyzed by GC/MS as described previously (Aktas et al., 2010). The other half was analyzed by LC/MS with an Agilent 1290 UPLC and an Agilent 6538 Accurate-Mass Q-TOF with a dual electrospray ionization (ESI) ion source. A 5-μL volume of each concentrated ethyl acetate solution was introduced to a ZORBAX SB-C18 column (2.1 mm × 100 mm, 1.8 μm). A gradient method was used for the separation (0–3 min 15% acetonitrile, 3–25 min linear gradient to 95% acetonitrile in water). The flow rate was 0.4 mL/min, and the temperature of the drying gas was maintained at 325°C. The data were analyzed using the Agilent B.04.00 MassHunter Qualitative Analysis software. A positive identification of key metabolites, such as alkylsuccinates, alkylmalonates, alkylbenzylsuccinates, and alkanoic acids, required that these were observed with the correct mass (±1 ppm), as well as with the retention times and MS/MS spectra observed for standard compounds.

## RESULTS

In total, we sequenced 191.6 Mb from three deep-sea sediment samples collected after the DWH blowout (**Table 1**), which included two sediment cores (GoM315 and GoM278) within 3 km of the DWH rig and one (GoM023) 127 km away (**Figure 1**). Post QC, 125.8 Mb were designated as high-quality sequences (252,082 individual reads), resulting in an average of 84,023 individual reads (average length of 491 bp/read) per deep-sea sediment core (**Table 1**).

**Table 1:** Data from the three GoM metagenomic libraries described in this study.

Features	GoM315	GoM278	GoM023
Distance from Deepwater Horizon blowout (km)	0.5	2.7	127.9
Depth below sea-level (m)	1,464	1,500	1,614
Basepairs sequenced prior to QC (Mb)	68.7	60.7	62.2
Individual reads prior to QC	144,700	127,356	122,703
Average length of reads prior to QC (bp)	474	476	506
Basepairs sequenced post QC (Mb)	43.9	38.8	41.1
Individual reads post QC	91,717	80,841	79,524
Average length of reads post QC(bp)	478	479	517
Prokaryotes	72,845	64,997	70,415
Eukaryotes	2,056	1,750	2,376
Viruses	268	372	74
Functional classifications (subsystems database)	59,175	52,599	55,130
Alpha diversity (species-level analysis)	1003.4	981.6	908.7
Chao 1 estimate ± SD (genus-level analysis)	593 ± 6.4	562 ± 2.6	582 ± 4.6
Shannon index (genus-level analysis)	5.64	5.55	5.55

### PHYLOGENETIC CLASSIFICATION

**FIGURE 2:**
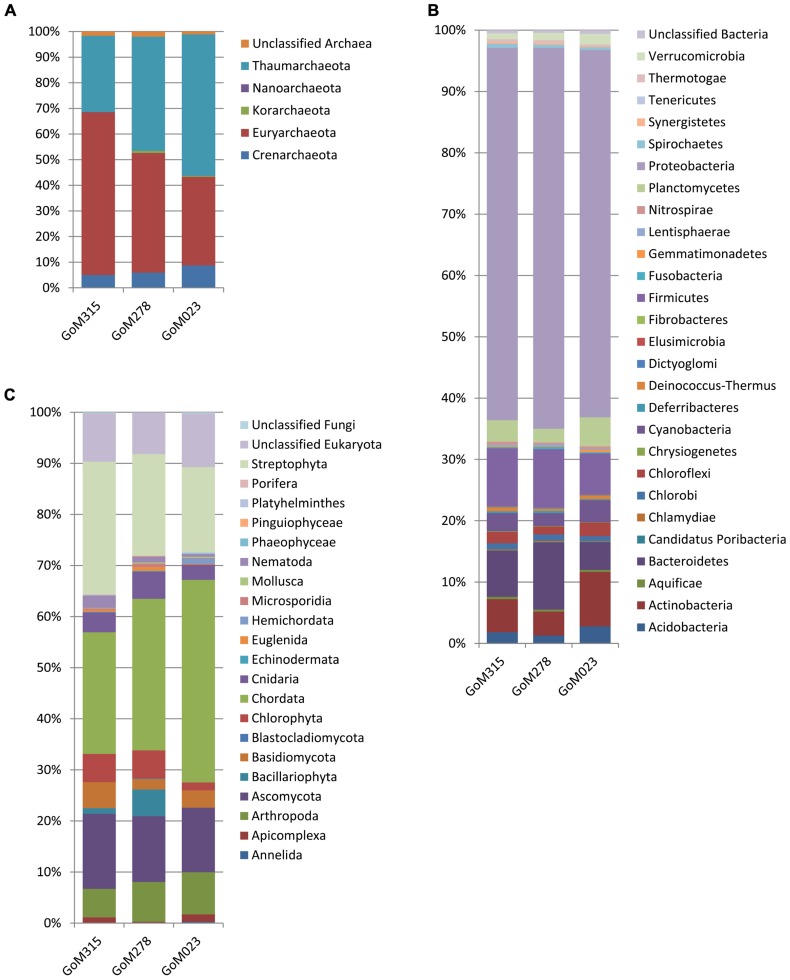
**Phylum-level organism classifications reveal differences among the three metagenomes sequenced in this study.** **(A)** Archaea; **(B)** bacteria; and **(C)** eukaryotes.

The MG-RAST classification tool revealed that at the domain level, all three samples had similar distributions. Bacteria (97–95%) dominated, while the archaea (4.2–2.2%) and eukaryotes (0.8–0.6%) contributed substantially less to the sediment communities. Differences among the three samples were observed when examined at the phylum level (**Figure 2**). The archaea associated with the deep-sea sediment cores were predominantly Euryarchaeota, Thaumarchaeota, and Crenarchaeota (**Figure 2A**). The Euryarchaeota dominated (65%) in the sample closest to the DWH rig (GoM315), but the same taxon and the Thaumarchaeota were equally represented (45%) at GoM278. The Thaumarchaeota dominated (55%) in the sample most distant from the spill site (GoM023).

Within the bacterial domain (**Figure 2B**), Proteobacteria dominated (60–65%) all three sediment cores, followed by Firmicutes in GoM315 (9%), Bacteroidetes in GoM278 (11%), and Actinobacteria in GoM023 (7%). The eukaryotic sequences represented 21 phyla from the Animalia, Fungi, Plantae, and Protista kingdoms. The Animalia phyla Arthropoda (e.g., crab and shrimp) and Chordata (e.g., fish and sharks) increased in abundance as the distance from the DWH rig increased, while the Cnidaria (e.g., corals and sponges) and Nematoda (e.g., roundworms) phyla were found only at greater abundance in the two sediment cores closest to the DWH rig. Although the number of viruses was relatively low (0.17–0.01%), a greater number of viruses were associated with the two samples located nearest the DWH rig (GoM315 and GoM278) compared to the sample furthest away (**Table 1**). Alpha diversity values calculated using annotated species-level distribution increased as the distance to the DWH rig lessened. However, other diversity indices revealed similar levels of both species in richness and diversity among the samples (**Table 1**).

**FIGURE 3:**
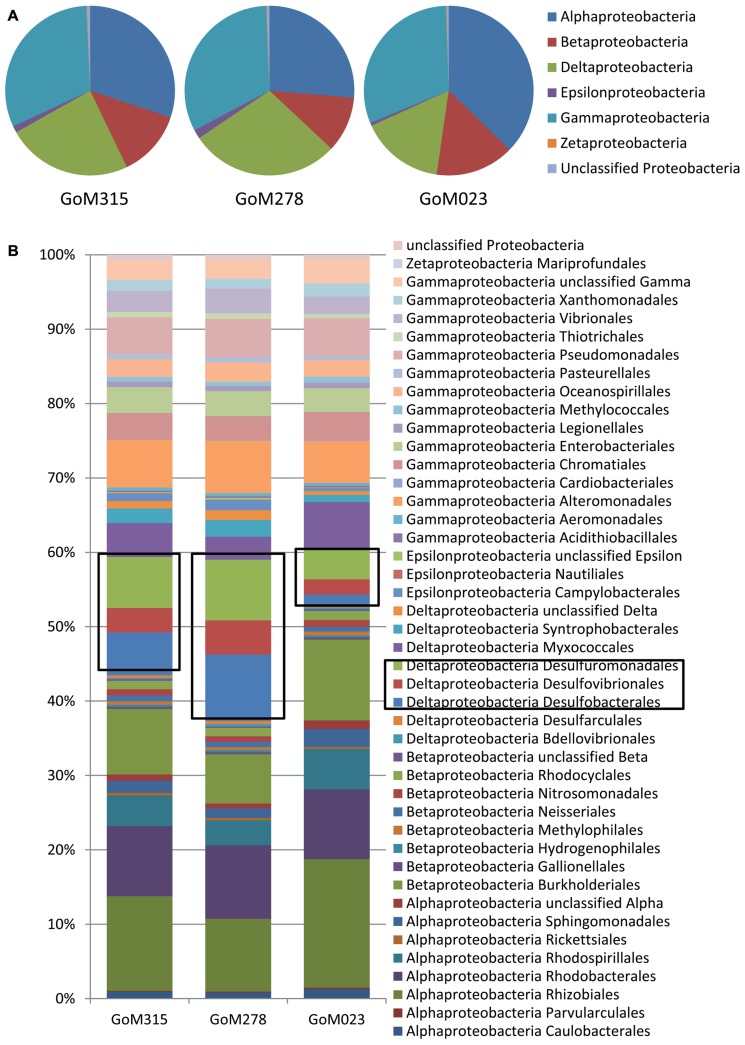
**Differences are observed among the sites closest to the DWH rig and the site located over a 100 km away when examining more of the Proteobacteria.** **(A)** Proteobacteria classes associated with each of the three sites reveals a decrease in the Deltaproteobacteria at the far site GoM023; **(B)** Proteobacteria order-level classifications identify Desulfobacterales, Desulfovibrionales, and Desulfuromonaldes as the major contributors to the difference observed.

The Proteobacteria associated with each sample were examined more closely in order to evaluate the potential for both aerobic and anaerobic oil biodegradation (**Figure 3**), since numerous Proteobacteria spp. are known to utilize petroleum hydrocarbons (Atlas, 1981; Widdel et al., 2010). The Gammaproteobacteria was the most diverse class with the *Shewanella*, *Marinobacter*, and *Pseudomonas* genera being the most common. Although the Gammaproteobacteria were similarly distributed (~33%), the distributions of both the Alphaproteobacteria and Deltaproteobacteria varied among the three deep-sea sediment samples (**Figure 3A**). The Alphaproteobacteria, predominantly the Rhizobiales and Rhodobacterales orders (**Figure 3B**), contributed to the highest percentage (37%) of Proteobacteria spp. in the sample furthest from the DWH rig (GoM023), while the two closer samples (GoM315 and GoM278) contained 30 and 26%, respectively. Greater numbers of sequences associated with GoM023 were detected in numerous Alphaproteobacteria genera, including *Rhizobium*, *Sinorhizobium*, * Bradyrhizobium*, * Roseobacter*, * Roseovarius*, and* Rhodobacter*. Deltaproteobacterial distributions revealed a wider range than the Gamma- and Alphaproteobacteria, one in which the two sediment cores closest to the DWH rig (GoM315 and GoM278) exhibited higher levels (26 and 30%, respectively), while the furthest core (GoM023) exhibited only 16% Deltaproteobacteria (**Figure 3A**). No single organism accounted for the shift in Deltaproteobacteria communities, rather a myriad of genera in the Desulfobacterales (e.g., *Desulfatibacillum*, *Desulfobacterium*, and *Desulfococcus*), Desulfovibrionales (e.g., *Desulfovibrio*), and Desulfuromonadales (e.g., *Geobacter*, and* Desulfomonas*) orders displayed higher levels in the GoM315 and GoM278 samples (**Figure 3B**).

### RECRUITMENT PLOTS

**FIGURE 4:**
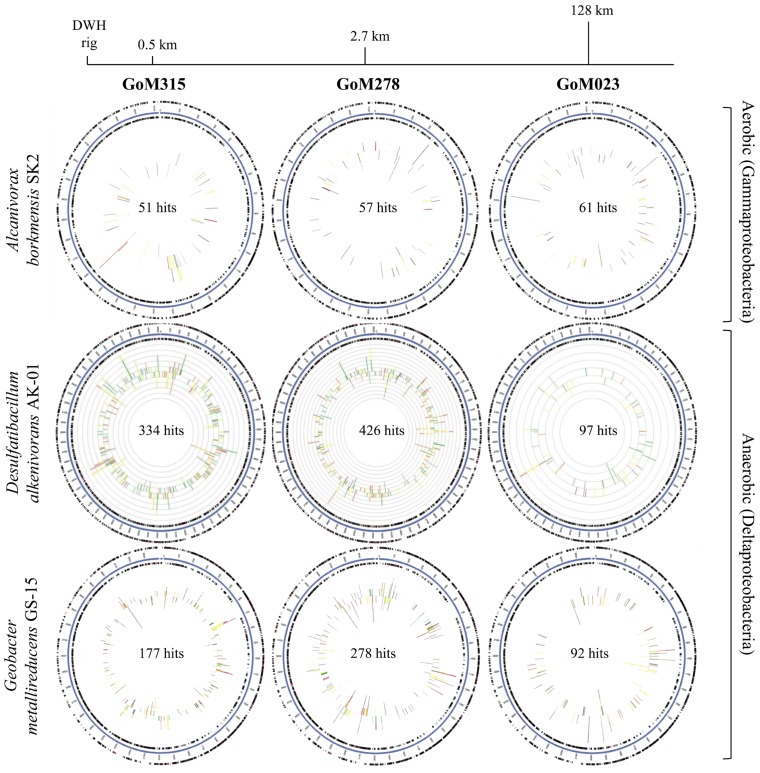
**Recruitment plots reveal an increased association with anaerobic hydrocarbon degraders in the deep-sea sediments near the DWH rig.** The blue circle represents the bacterial contigs for the genome of interest; while the two black rings map genes on the forward and reverse strands. The inner graph consists of two stacked bar plots representing the number of matches to genes on the forward and reverse strands. The bars are color coded according to the e-value of the matches with red (<1e - 30), orange (1e - 30 to 1e - 20), yellow (1e - 20 to 1e - 10), and green (1e - 10 to 1e - 5).

Recruitment plots, comparing sequences from each metagenomic library to the genomes of specific organisms, supported the presence of known hydrocarbon-utilizing Proteobacteria (**Table 2**). The analysis revealed a total of 169, 857, and 547 sequences, respectively, matching to features of the *Alcanivorax borkumensis *SK2 genome (Proteobacteria, Gammaproteobacteria, Oceanospirillales, Alcanivoracaceae; Yakimov et al., 1998; Schneiker et al., 2006), the *Desulfatibacillum alkenivorans* AK-01 genome (Proteobacteria, Deltaproteobacteria, Desulfobacterales, Desulfobacteraceae; So and Young, 1999; Callaghan et al., 2012), and the *G. metallireducens* GS-15 genome (Proteobacteria, Deltaproteobacteria, Desulfuromonadales, Geobacteraceae; Lovley et al., 1993) in all three deep-sea sediment samples. Interestingly, matches to the aerobic hydrocarbon degrader, *Alcanivorax borkumensis *SK2 (51–61 sequence hits), remained consistent among all three samples; whereas, the comparison to the two anaerobic hydrocarbon degraders, *Desulfatibacillum alkenivorans* AK-01 (97–426 sequence hits) and *G. metallireducens* GS-15 (92–278 sequence hits), revealed a greater number of sequence matches to the two samples (GoM315 and GoM278) closest to the DWH well (**Figure 4**). Similarly, sequences recruited to *Desulfococcus oleovorans* Hxd3 (**Table 2**), a model sulfate-reducing alkane/alkene utilizer, in all three samples; however, GoM315 and GoM278 recruited a greater number of sequences (256 and 332, respectively) compared to GoM023 (79).

**Table 2:** Top ranked recruitment results for each of the GoM deep-sea sediment metagenomic libraries.

	Recruited genome	Rank	Number of sequences	Number of features	Features in genome	Genome coverage(%)
GoM315	*Desulfobacterium autotrophicum* HRM2	1	464	428	4943	8.66
	“Candidatus” *Solibacter usitatus* Ellin6076	2	397	300	7826	3.83
	*Desulfatibacillum alkenivorans* AK-01	3	334	288	5252	5.48
	*Nitrosopumilus maritimus* SCM1	4	268	223	1796	12.42
	*Desulfococcus oleovorans* Hxd3	5	256	227	3265	6.95
	*Rhodopirellula baltica* SH 1	7	211	193	7325	2.63
	*Desulfotalea psychrophila* LSv54	12	185	169	3234	5.23
	*Haliangium ochraceum* DSM14365	15	174	155	6719	2.31
	*Cenarchaeum symbiosium* A	34	122	111	2017	5.5
	*Archaeoglobus fulgidus* DSM4304	>300	20	18	2420	0.74
GoM278	*Desulfobacterium autotrophicum* HRM2	1	746	593	4943	12
	*Desulfatibacillum alkenivorans * AK-01	2	426	362	5252	6.89
	*Nitrosopumilus maritimus * SCM1	3	358	294	1796	16.37
	*Desulfococcus oleovorans* Hxd3	4	332	288	3265	8.82
	*Desulfotalea psychrophila* LSv54	5	256	221	3234	6.83
	“*Candidatus” Solibacter usitatus* Ellin6076	7	218	189	7826	2.42
	*Cenarchaeum symbiosium* A	10	187	165	2017	8.18
	*Haliangium ochraceum* DSM14365	20	134	123	6719	1.83
	*Rhodopirellula baltica* SH 1	22	124	124	7325	1.69
	*Archaeoglobus fulgidus* DSM4304	>300	33	31	2420	1.28
GoM023	*Nitrosopumilus maritimus* SCM1	1	713	520	1796	28.95
	“*Candidatus” Solibacter usitatus* Ellin6076	2	564	410	7826	5.24
	*Cenarchaeum symbiosium* A	3	373	296	2017	14.68
	*Haliangium ochraceum* DSM14365	4	290	242	6719	3.6
	*Rhodopirellula baltica* SH 1	5	279	243	7325	3.32
	*Desulfatibacillum alkenivorans* AK-01	58	97	81	5252	1.54
	*Desulfococcus oleovorans* Hxd3	79	81	74	3265	2.27
	*Desulfobacterium autotrophicum* HRM2	102	72	70	4943	1.42
	*Desulfotalea psychrophila* LSv54	>200	39	37	3234	1.14
	*Archaeoglobus fulgidus* DSM4304	>300	16	14	2420	0.58

### FUNCTIONAL GENE ANALYSIS

**FIGURE 5:**
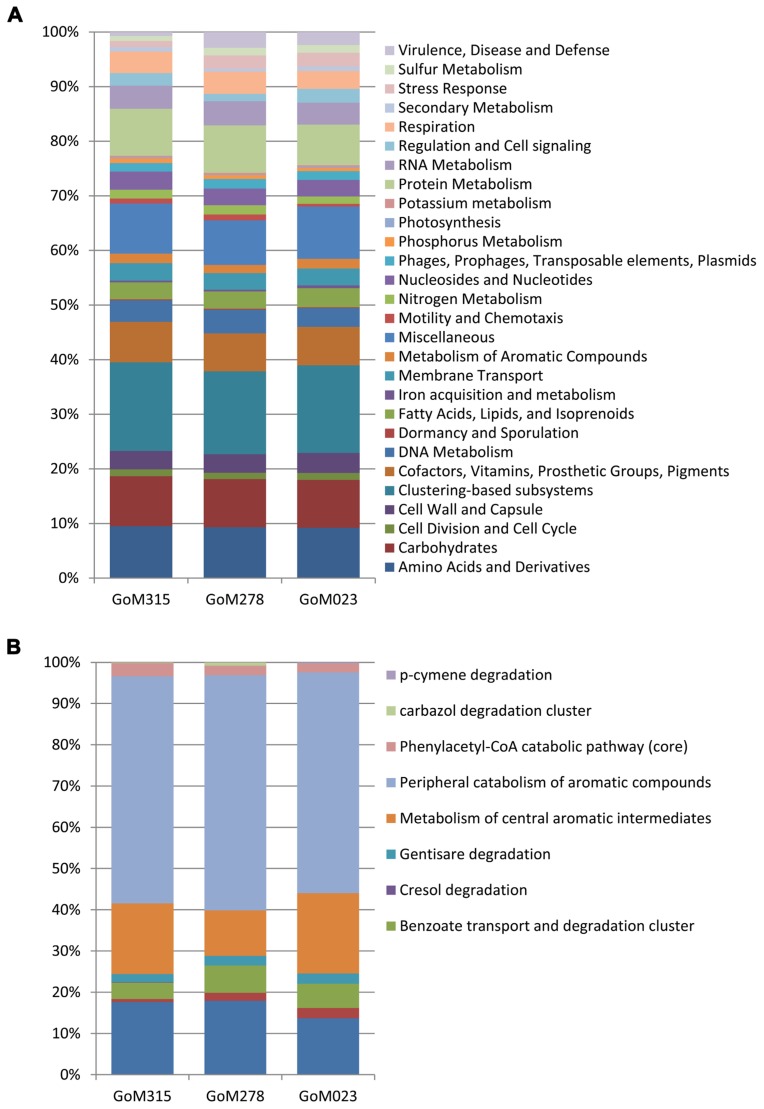
**Functional classifications of the metagenomic sequences.** **(A)** Similar functional fingerprints are observed at the broadest subsystem classification. **(B)** Functional genes associated with the “metabolism of aromatic compounds” reveal a decreased association with “anaerobic degradation in aromatic compounds” in GoM023.

All three samples revealed a similar functional blueprint at the broadest level of classification (**Figure 5A**). Genes coding for clustering-based subsystems (15–16%), amino acid and derivatives (9.2–9.3%), miscellaneous (8.2–9.5%), carbohydrates (8.8%), and protein metabolism (7.4–8.7%) represented the five most abundant categories when classified using the SEED database (**Figure 5A**). Analysis using COG classifications revealed a similar functional distribution, with the majority of sequences assigned to metabolism (45–46%), followed by cellular processes and signaling (19–21%), information storage and processing (17–18%), and poorly characterized categories (15–18%). There was genetic evidence in all three samples for the potential degradation of oil compounds, including genes vital to both the aerobic (e.g., mono- and dioxygenases) and anaerobic degradation (e.g., *bss* and benzoyl-CoA reductase) of compounds such as butyrate, benzoate, toluene, and alkanoic acids (Table S1 in Supplementary Material). Functional analysis of the “metabolism of aromatic compounds” subsystem provided additional evidence of a greater potential for anaerobic metabolism in the two samples nearest the DWH rig compared to the more distant sample (**Figure 5B**). GoM315 (located 0.5 km from the DWH rig) exhibited the highest percentage (15%) of anaerobic degradation genes for aromatic compounds, while GoM023 (located 128 km from the DWH rig) exhibited the lowest (9.9%). Notably, the metagenomics data revealed *bssA* in GoM315 only, the sample closest to the DWH well, and the complete complement (subunits D–G) of benzoyl-CoA reductase genes (Egland et al., 1997) was detected in GoM315 and GoM278, but not GoM023, the site farthest from the DWH well.

### CLONE LIBRARIES

**FIGURE 6:**
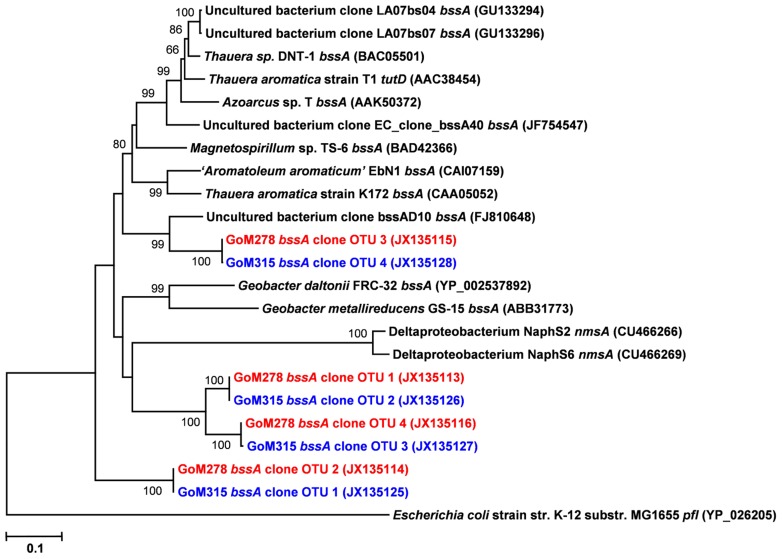
**Neighbor-joining dendrogram of *bssA *clone sequences obtained from GoM sediments (GoM278 – red; GoM315 – blue) compared to *bssA* sequences of reference strains and BLAST matches.** The tree was constructed using the Tajima–Nei distance method (scale bar), with pairwise deletion and performing 10,000 bootstrap replicates. Bootstrap values below 65 are not shown. pyruvate formate lyase (*pfl*) served as the outgroup. Numbers in parentheses represent NCBI GenBank accession numbers. *bss*, benzylsuccinate synthase; *tut*, toluene-utilizing (i.e., benzylsuccinate synthase); *nms*, naphthylmethylsuccinate synthase.

**FIGURE 7:**
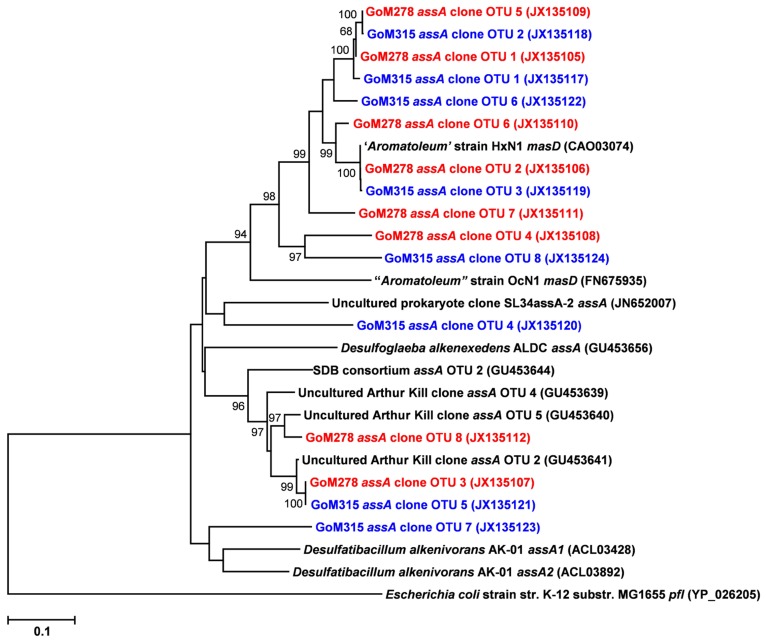
**Neighbor-joining dendrogram of *assA *clone sequences obtained from GoM sediments (GoM278 – red; GoM315 – blue) compared to *assA*/*masD* sequences of reference strains and BLAST matches.** The tree was constructed using the Tajima–Nei distance method (scale bar), with pairwise deletion and performing 10,000 bootstrap replicates. Bootstrap values below 65 are not shown. Pyruvate formate lyase (*pfl*) served as the outgroup. Numbers in parentheses represent NCBI GenBank accession numbers. *ass*, alkylsuccinate synthase; *mas*, methylalkylsuccinate synthase.

Functional gene libraries supported the metagenomic analysis and also suggested a greater genetic potential for anaerobic hydrocarbon degradation at the two sites near the DWH well, with respect to the *assA* and *bssA* genes. The *assA* and *bssA* genes encode the catalytic subunits of the glycyl radical enzymes, ASS, MAS; Callaghan et al., 2008; Grundmann et al., 2008) and BSS; Leuthner et al., 1998), respectively. Based on previous studies, ASS/MAS presumably catalyzes the addition of *n*-alkanes to fumarate (Callaghan et al., 2008; Grundmann et al., 2008) to form methylalkylsuccinic acids (for review see Widdel and Grundmann, 2010), whereas BSS catalyzes the addition of aromatic hydrocarbons to fumarate to yield benzylsuccinic acids and benzylsuccinate derivatives (for review see Boll and Heider, 2010). Both *assA* and *bssA* have been used as biomarkers, in conjunction with metabolite profiling, as evidence of *in situ* aliphatic and aromatic hydrocarbon degradation (Beller et al., 2008; Callaghan et al., 2010; Yagi et al., 2010; Oka et al., 2011; Wawrik et al., 2012). Of the nine primer sets tested (Callaghan et al., 2010), primer set 2 (specific to *bssA*) yielded four *bssA* OTUs in GoM278 sediment and four *bssA* OTUs in GoM315 sediment (**Figure 6**). Primer set 7 (specific to *assA*) yielded eight *assA* OTUs in GoM278 and eight *assA* OTUs in GoM315 (**Figure 7**). A comparison of the *bssA* and *assA* OTU sequences revealed that there are unique and shared OTUs between the two sites. Sequence identities ranged from 68.8 to 100% and 63.7 to 100% for *bssA* and *assA*, respectively. Based on BlastX and BlastN, the GoM *bssA *clone sequences were similar to those from uncultured bacteria as well as to *bssA* in *Thauera aromatica* K172 and *Azoarcus* sp. T (Table S2 in Supplementary Material). Based on BlastX and BlastN, the GoM *assA *clone sequences were similar to those from uncultured bacteria, as well as to *masD* in “*Aromatoleum*” sp. HxN1 (Table S2 in Supplementary Material). The *assA* and *bssA* genes were not detected in sediment collected from the background site, GoM023, under the PCR conditions and primers tested in this study.

### METABOLITE PROFILING

**FIGURE 8:**
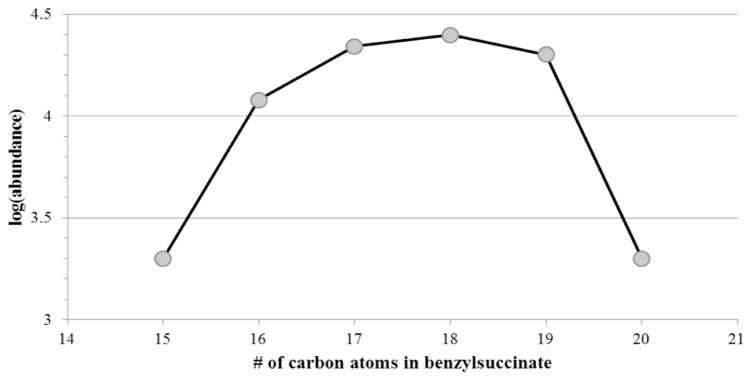
**Logarithm of abundance of benzyl succinates in the sample GoM315**.

We specifically looked for the presence of alkylsuccinate derivatives that were presumed metabolites formed by the addition of hydrocarbon substrates across the double bond of fumarate (Biegert et al., 1996; Kropp et al., 2000; Elshahed et al., 2001; Gieg and Suflita, 2005). For example, the presence of benzyl- or alkyl-succinic acids indicates the anaerobic metabolic decay of alkylated aromatic or *n*-alkane hydrocarbons, respectively (Davidova et al., 2005; Duncan et al., 2009; Parisi et al., 2009). Straight chain alkanes and alkenes with carbon lengths from C11 to C14 and from C13 to C22, respectively, were detected using GC/MS in the two sites closest to the spill site (GoM278 and GoM315). A few branched alkanes and alkenes were also observed. *n*-Alkane and *n*-alkene hydrocarbons were not detected in the background sample (GoM023). With GC/MS, alkanoic acids in GoM278 (2.7 km) with lengths between C14 and C18 were detected, whereas the lengths ranged from C7 to C22 in GoM315 (0.5 km). Alkylsuccinate or alkylmalonate metabolites typically associated with the anaerobic biodegradation of *n*-alkanes via “fumarate addition” were below detection levels in all samples. However, putative benzylsuccinates were identified in the samples, based on their metastable fragmentation pattern of ≈5% loss of CO_2_ and no detectable loss of H_2_O in MS mode. The highest abundances were observed for C16 to C19 benzylsuccinates (**Figure 8**), and their abundances were also three times higher in GoM315 (0.5 km) than in the other two samples. The presence of benzylsuccinates is consistent with the detection of *bssA* genotypes. Benzoate, a central metabolite of both aerobic and anaerobic hydrocarbon metabolism, was also detected in the two samples closest to the spill site.

### COMPARATIVE METAGENOMICS

**FIGURE 9:**
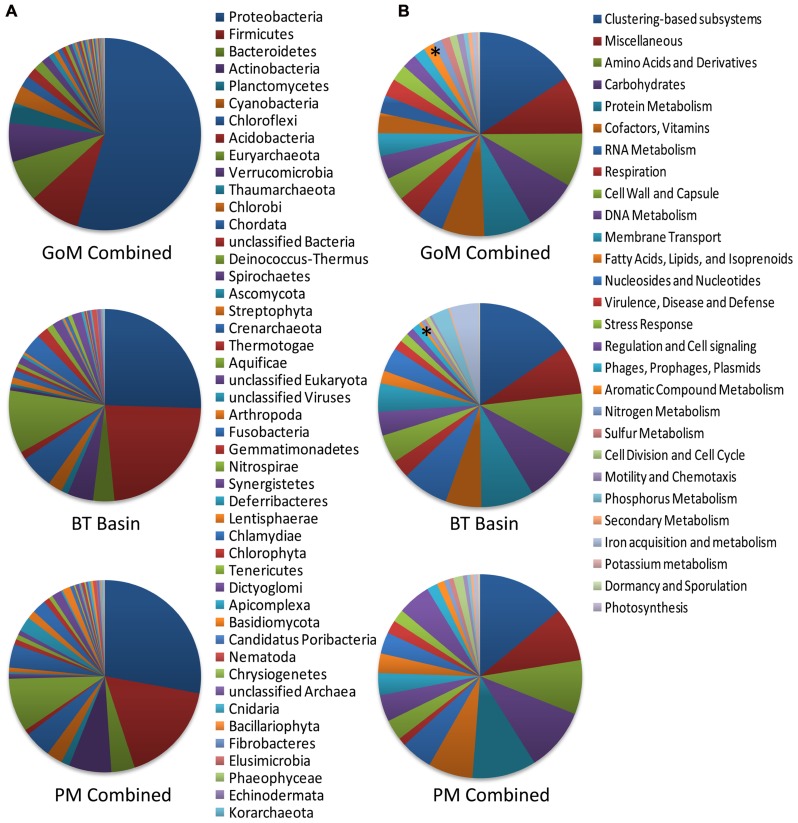
**Phylogenetic and functional comparison of deep-sea sediment metagenomes across three studies.** **(A)** Phylogenetic comparison at the phylum level reveals differences among this study and the two previously performed by Biddle et al. (2008, 2011). **(B)** Functional comparison of the broadest level of subsystem classifications reveals a more similar pattern between the three studies. Asterisk ^“*”^ denotes the “aromatic compound metabolism” category; GoM combined, represents the collective data from GoM315, GoM278, and GoM023; PM combined, represents the collective data from PM01^*^, PM01, and PM50. The BT basin represents a single metagenome.

**FIGURE 10:**
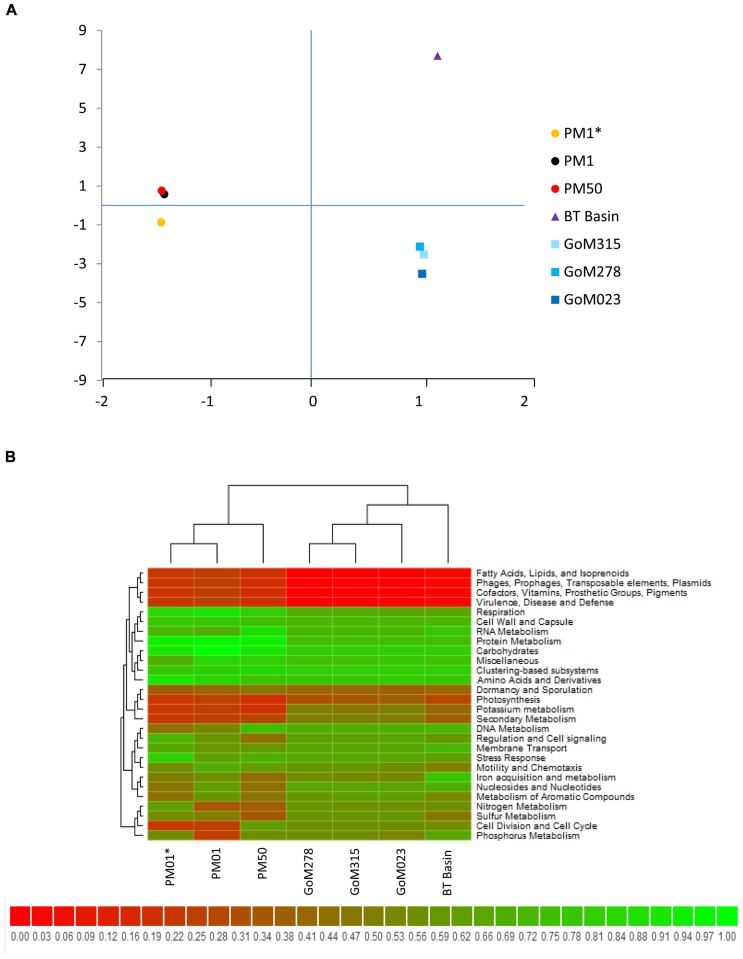
**Cross-study comparisons of deep-sea metagenomes.** **(A)** Principal component analysis using organism classifications (species-level) from the current study as well as those from two studies (Biddle et al., 2008, 2011) reveals clear geographic separation, in addition to a second component of separation that is currently unknown. **(B)** Hierarchical clustering combined with heat mapping based on subsystem classifications reveals similar partitioning among the three studies compared.

Comparison of our metagenomic data to that of two other deep-sea metagenomes revealed a number of interesting differences. The first metagenomic study examined deep-subsurface sediment cores (PM01*, PM01, PM50) from the nutrient-rich area of the Peru Margin (Biddle et al., 2008), while the second examined an oligotrophic subsurface sediment core from the Gulf of Mexico (BT Basin) prior to the DWH blowout (Biddle et al., 2011). In both studies the samples were subsurface sediments collected at a depth of two meters or greater, whereas the samples collected in this study were surficial samples collected at the interface between the water and the sediment. Distributions of organisms at the domain level were slightly different between the Peru Margin/BT Basin samples and our GoM samples, with the former harboring a greater percentage of archaea (18.1–8.6% compared to 2.9–3.3%) and eukaryotes (17.7–5.8% compared to 2.6–3.3%). At the phylum level, the Peru Margin and BT Basin data revealed a different picture from this study with a more even distribution of Proteobacteria and Firmicutes, followed by Euryarchaeota and Chloroflexi (**Figure 9A**). Although the functional gene patterns were similar among the three studies, sequences associated with the “metabolism of aromatic compounds” category were more abundant in all three of our samples (1.4–1.9%) following the DWH oil spill compared to the BT Basin (0.5%) level evaluated prior to the spill (**Figure 9B**). Hierarchical clustering analysis, based on subsystem functional classification, revealed geographical separation between the Peru Margin and Gulf of Mexico samples (**Figure 10A**). Within the Gulf of Mexico cluster, the BT Basin clustered separately from GoM023, GoM278, and GoM315. Furthermore, GoM315 and GoM278, the samples located relatively close to the DWH rig, clustered separately from GoM023, the sample furthest from the DWH rig. A similar pattern of separation was visualized using principal component analysis (**Figure 10B**) with the organism classifications.

## DISCUSSION

In this study, we present three new metagenomic data sets from deep-sea sediments of the Gulf of Mexico following the DWH oil spill. Due to logistical and political circumstances surrounding the DWH oil spill, three samples were the extent of which we were able to obtain. These data, however, present a unique opportunity to examine deep-sea sediments following a massive anthropogenic hydrocarbon loading event and triples the number of metagenomic datasets previously available (one metagenome; Biddle et al., 2011) for deep-sea subsurface sediments in the Gulf of Mexico. Although the lack of replication makes it difficult to draw wide conclusions regarding the effects of hydrocarbon exposure on microbial community composition and activity, these metagenomes provide important data to make baseline observations that will need to be examined more thoroughly in future studies.

Two previous deep-sea metagenomic studies resulted in the suggestion that there is a core metagenomic structure for deep-sea sediments, composed of four main microbial groups (Euryarchaeota, Proteobacteria, Firmicutes, and Chloroflexi) that can vary depending on specific parameters, such as depth, organic carbon content, and geography (Biddle et al., 2008; Biddle et al., 2011). These four microbial taxa were also detected in GoM sediment samples in the present work; however, they do not constitute the four major groups detected in this report (Proteobacteria, Bacteroidetes, Firmicutes, and Actinobacteria). Despite similarities to the microbial communities described previously (Biddle et al., 2008; Biddle et al., 2011), our cross-study comparisons via hierarchical clustering and principal component analyses reveal a distinct separation between the Peru Margin communities (Biddle et al., 2008) and both Gulf of Mexico communities (Biddle et al., 2011, and current report). This is of particular interest, since the two previous studies were subsurface samples, compared to our surficial samples. A metagenomic fosmid library of deep-sea sediments from the organic-rich Qiongdongnan Basin in the South China Sea (Hu et al., 2010) revealed a community structure that was more similar to this study than to those of the Biddle et al. (2008), 2011 studies. These data suggest the possibility that organic carbon content is more relevant to microbial community structure than geography. Hu et al. (2010) detected Proteobacteria as the dominant (~43%) bacterial phylum, and Deltaproteobacteria as the most abundant class within this phylum. KEGG analysis of the fosmid ends also revealed genes associated with the biodegradation pathways of numerous xenobiotics including, but not limited to dichloroethane, benzoate, biphenyl, ethylbenzene, fluorene, naphthalene, anthracene, styrene, tetrachloroethene, and gamma-hexachlorocyclohexane. The detection of genes related to the biodegradation of xenobiotic compounds in their study and ours supports the premise that deep-sea sediment microbial communities have the potential to metabolize a diverse array of organic compounds, including many that are found in oil.

Numerous bacterial species have evolved the ability to metabolize aliphatic (e.g., alkanes and alkenes) and aromatic hydrocarbons (e.g., mono- and polynuclear), with the most rapid and complete degradation achieved through aerobic processes (Fritsche and Hofrichter, 2008). The majority of characterized oil-degraders within marine systems are aerobic members of the Alpha- and Gammaproteobacteria (for reviews see Head et al., 2006; Kim and Kwon, 2010) that use mono- and dioxygenases to initiate degradation (Haddock, 2010; Rojo, 2010). This includes *Alcanivorax borkumensis*, a ubiquitous gammaproteobacterium in marine environments, which is known to utilize aliphatic hydrocarbons (Yakimov et al., 1998; Schneiker et al., 2006; dos Santos et al., 2010). Following the DWH blow out, *Oceanospirillales* was shown to be the dominant bacterial orders associated with the resulting deepwater (~1100 m) oil plume (i.e., more than 90% of the sequences were classified as* Oceanospirillales*, predominantly from one monophylectic lineage(Hazen et al., 2010). Our metagenomic analysis revealed the presence, in all three deep-sea sediment libraries, of bacteria belonging to the broader *Oceanospirillales* order, including 51–61 sequences specifically recruited to the *Alcanivorax borkumensis* genome in each sample (**Figure 4**). The abundance of *Oceanospirillales*, however, was relatively low (< 2% of bacterial sequences) compared to those found in the deep water oil plume (Hazen et al., 2010; Mason et al., 2012). GoM315, GoM278, and GoM023 exhibited similar levels of *Oceanospirillales* spp. (1.5, 1.7, and 1.4% of bacterial sequences, respectively) and Gammaproteobacteria in general (19, 20, and 19% of bacterial sequences, respectively), showing no correlation to the hydrocarbon levels associated with each sample. Alphaproteobacteria associated with aerobic oil-degradation were also found at very low abundances with similar levels across the deep-sea sediment samples, including *Roseovarius* spp. and *Maricaulis *spp. (<0.5 and <0.3% of bacterial species, respectively). Nonetheless, mono- and dioxygenases were present in all three samples. These data indicate that the potential for aerobic degradation is present in these samples, albeit at much lower levels than observed in the water column (Hazen et al., 2010; Mason et al., 2012), and that the level of hydrocarbon exposure did not significantly impact this potential. One potential explanation for this is that the hydrocarbons susceptible to aerobic degradation were depleted rapidly in the water column, either by dispersants or by the quick responding bacterial blooms of aerobic hydrocarbon-degrading microorganisms (Hazen et al., 2010; Kessler et al., 2011). As a result, the hydrocarbon loading that occurred in the deep-sea sediments may not have promoted the growth of microorganisms capable of aerobic hydrocarbon degradation, but rather that of microorganisms capable of degrading the remaining recalcitrant hydrocarbons that require anaerobic processing. It is also possible, however, that we sampled at a time when the community was just beginning to shift to reflect the increasing importance of anaerobic microbes. Future work involving time series samples and/or the analysis of aerobic metabolites will be necessary to provide further insights.

Anaerobic biodegradation of hydrocarbons is an important biogeochemical process in a variety of deep-subsurface environments (Aitken et al., 2004; Jones et al., 2007; Wawrik et al., 2012). Studies during the last two decades have highlighted the ability of anaerobic microorganisms to metabolize a variety of hydrocarbons, including *n*-alkanes, *n*-alkenes, alicyclic hydrocarbons, and mono- and polycyclic aromatic compounds (for reviews see Boll and Heider, 2010; Widdel and Grundmann, 2010; Widdel et al., 2010). To date, the most well-characterized anaerobic mechanism for hydrocarbon activation and degradation is via addition of the hydrocarbon to the double bond of fumarate (“fumarate addition”) catalyzed by glycyl radical enzymes (for reviews see Boll and Heider, 2010; Widdel and Grundmann, 2010). Deltaproteobacteria, in particular, have been implicated in “fumarate addition” of both aromatic and aliphatic hydrocarbons (Widdel et al., 2010). In this study, metagenomic analysis revealed an increase in the percentage of bacterial sequences that represent Deltaproteobacteria associated with the sediment cores closest to the DWH well, where there were higher levels of PAHs (Operational Science Advisory Team, 2010) and detectable levels of alkanes and alkenes. It should be noted that the increase in Deltaproteobacteria is potentially an indirect effect of the increased dead biomass from the oil spill, which cannot be ruled out by this study. In any case, recruitment plots demonstrated that 857 and 547 of the metagenomic sequences mapped onto the Deltaproteobacterial genomes of *Desulfatibacillum alkenivorans* AK-01 and *G. metallireducens* GS-15, respectively. The increases in Deltaproteobacteria were also concurrent with an increase in functional genes involved in the anaerobic degradation of hydrocarbons, such as BSS, acetyl-CoA acetyltransferase and benzoyl-CoA reductase. These results suggest that the microbial response to anthropogenic hydrocarbon loading may mirror aspects of microbial communities associated with Gulf of Mexico natural seeps, where Deltaproteobacteria play a dominant role in their biogeochemical activity, including anaerobic hydrocarbon degradation (Lloyd et al., 2010; Orcutt et al., 2010). Most likely, however, the specific genus-level lineages of Deltaproteobacteria will be dependent on the hydrocarbon source present, since the natural gas-rich seeps contain specialized deltaproteobacterial groups for anaerobic methane utilization that are unlikely to thrive in sediments with more recalcitrant oil remnants.

Clone libraries of *assA* and *bssA* supported the metagenomic analysis. Both genotypes were detected in sediments near the DWH well (GoM278 and GoM315), but not at the unimpacted site (GoM023). The presence of *assA* and *bssA* suggests the potential for both aliphatic and aromatic hydrocarbon activation via “fumarate addition.” Although *assA* genotypes were detected in sediments from GoM278 and GoM315, alkylsuccinates were not detected in these samples. However, this should not be interpreted as conclusive evidence that aliphatic substrates were not being metabolized. The requisite metabolites are usually in low abundance (typically nM) and transitory and could have easily been further metabolized or been below method detection limits. The alkanoic acid compounds detected in the GoM samples could have been formed via multiple biological pathways, including aerobic and anaerobic transformation of aliphatic hydrocarbons, but they are not highly diagnostic. Despite the non-detection of alkylsuccinates, both *bssA* genotypes *and *the putative benzylsuccinate metabolites were detected in the two sediment cores closest to the spill site, suggesting *in situ* anaerobic biodegradation of alkylbenzenes. This is consistent with the increased number of genes related to “aromatic metabolism” detected in the corresponding sediments via metagenomic analysis. Benzoate was also detected in GoM278 and GoM315 sediments, suggesting further transformation of the benzylsuccinate derivatives of monoaromatic hydrocarbons (Beller and Spormann, 1997; Leuthner et al., 1998). However, benzoate can be formed during the metabolism of a wide variety of aromatic compounds under aerobic *and* anaerobic conditions.

Overall, this study took an interdisciplinary approach of investigating the phylogenetic composition and functional potential of Gulf of Mexico deep-sea sediment communities following the DWH oil spill. Based on metagenomic analyses, functional gene clone libraries, and metabolite profiling, the data herein suggest that the presence of PAHs, alkanes, and alkenes may influence the microbial community through the enrichment of Deltaproteobacteria capable of anaerobic hydrocarbon metabolism. This evidence suggests that the microbial communities exposed to anthropogenic hydrocarbon loading in the Gulf of Mexico deep-sea sediments likely impacted the bioremediation of the DWH oil spill through anaerobic degradation, which has been previously overlooked. The integrated approach used herein augments other efforts to deduce the fate of the oil spilled in the DWH incident and to assess the impact of the spill on the indigenous microbial communities.

## Conflict of Interest Statement

The authors declare that the research was conducted in the absence of any commercial or financial relationships that could be construed as a potential conflict of interest.

## SUPPLEMENTARY MATERIAL

The Supplementary Material for this article can be found online at: http://www.frontiersin.org/Microbiological_Chemistry/10.3389/fmicb.2013.00050/abstract
